# Live Images of Donor Dendritic Cells Trafficking *via* CX3CR1 Pathway

**DOI:** 10.3389/fimmu.2016.00412

**Published:** 2016-10-14

**Authors:** Takuya Ueno, Pilhan Kim, Martina M. McGrath, Melissa Y. Yeung, Tetsunosuke Shimizu, Keehoon Jung, Mohamed H. Sayegh, Anil K. Chandraker, Reza Abdi, Seok H. Yun

**Affiliations:** ^1^Renal Division, Transplantation Research Center, Brigham and Women’s Hospital, Harvard Medical School, Boston, MA, USA; ^2^Department of Dermatology, Wellman Center for Photomedicine, Massachusetts General Hospital, Harvard Medical School, Boston, MA, USA

**Keywords:** transplantation, imaging, chemokine, CX3CR1, dendritic cells

## Abstract

**Background:**

A number of studies have demonstrated the role of CX3CR1 in regulating the migration of monocytes into peripheral tissue and their transformation into dendritic cell (DC). No data are yet available on the importance of chemokine pathways in regulating homeostasis of DC in heart transplants. Recently, we showed that recipients of heart allografts from CX3CR1^−/−^ donors show longer survival. To assess the trafficking of dDC, we have developed and tested a novel *in vivo* imaging tool in CX3CR1^GFP/+^ DC (B6 background) heart graft into BALB/c recipient model.

**Results:**

Majority of GFP^+^ cells were noted in the middle of cardiac myocyte. However few hours post transplant, they experienced morphological changes including stretching their extensions (3 and 24 h). However, images from 72 h at cardiac graft showed many of GFP^+^ cells moved to vessel areas. GFP^+^ cells were detected in near vessel wall. Only one GFP^+^ cell was observed in three lymph nodes (two mesenteric and one inguinal) (72 h).

**Conclusion:**

Our data indicate that immediately post transplant dDC undergo morphological changes and traffic out of the organs via systemic circulation. While, we still noted presence of dDC in the transplanted organs, their trafficking to lymphoid tissue remains to be fully explored.

## Introduction

Organ transplantation is a life-saving procedure for patients with end-stage organ failure. The introduction of novel immunosuppressive drugs has led to significant improvement in short-term survival rates of solid organ allografts. Nevertheless, these drugs result in increased rates of cancer and opportunistic infections. Furthermore, they do not prevent chronic allograft dysfunction, the leading cause of graft failure after 1 year posttransplantation. To improve transplantation outcomes, it is critical to continue the development of novel strategies to prevent acute and chronic rejection. To accomplish this goal, it is necessary to elucidate the exact basic mechanisms leading to acute and chronic rejection, and tolerance. It is clear that T cells play a central role in the process of allograft rejection. However, donor dendritic cells (dDC) are the most potent antigen-presenting cells and have proven to be major regulators in determining the fate of the alloimmune response. The ability of dDC to mount an efficient alloimmune response relies on their capacity to be recruited in the donor tissue and their ability to traffic to secondary lymphoid tissues in the recipients, both of which are tightly controlled by specific chemokine pathways.

**Figure 1:**
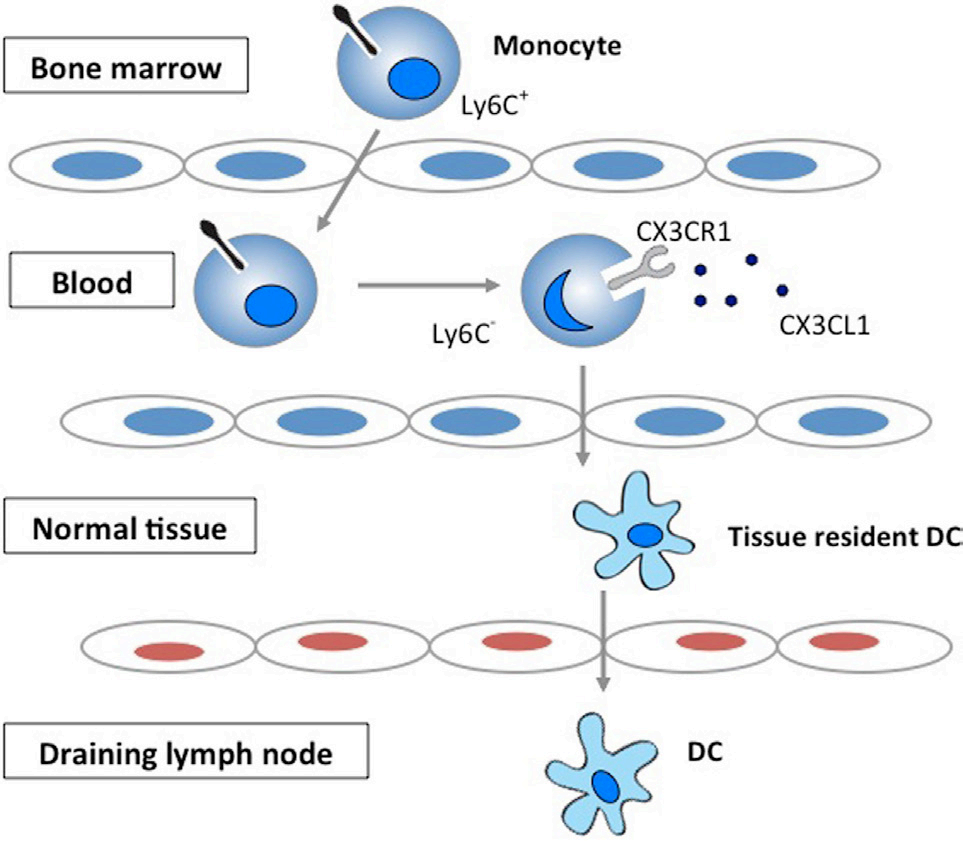
**The role of CX3CR1 signaling in the generation of lymphoid tissue DC**. Development and function of monocyte subsets in mice. In the absence of inflammation, CX3CR1^hi^Ly6C^−^ monocytes enter the tissues and replenish the tissue-resident macrophage and DC populations.

Chemokines play important roles in selectively recruiting leukocytes to the site of inflammation as well as in the activation of leukocytes following recruitment (1–3). Chemokines have also been increasingly recognized for their role in regulating migration of circulating monocytes and their transformation to tissue dendritic cells (DCs). The CX3CR1 signaling has been demonstrated to be important in the generation of lymphoid tissue DC by regulating the trafficking of circulating monocytes into tissue (4–6) (Figure 1).

Our specific hypothesis is that the CX3CR1 signaling plays an important role in the seeding of dDC in heart tissue as well as their function posttransplantation, hence playing a key role in directing the alloimmune response toward rejection and tolerance. Based on advances in cellular imaging, it is now feasible to follow some of these key cellular and molecular events *in vivo* in the beating heart (7, 8). We propose to study the chemokine-mediated regulation of dDC function and trafficking to recipient lymphoid tissues and transplanted graft.

## Materials and Methods

### Mice

We performed our experiments using Itgax-DTR/GFP57Lan (DTR-GFP-DC) and CX3CR1GFP/GFP mice. GFP is linked to the CD11c promoter and CX3CR1 promoters, respectively (9). These mice will be used as donors of heart transplants, thus providing a well refined and novel system to study dDC and the role of CX3CR1 signaling in dDC trafficking posttransplantation. BALB/c (H-2^d^) (WT) mice were purchased from the Jackson Laboratory (Bar Harbor, ME, USA). Mice were maintained in accordance with Harvard Medical School institutional guidelines and used at 6–10 weeks of age.

### Heterotopic Heart Transplantation

Vascularized heart allografts were transplanted intra-abdominally using microsurgical techniques as we described (10). We used a murine cardiac transplant model: CX3CR1^GFP/+^ DC (B6 background) heart graft into BALB/c fully allogenic recipient mice.

### *In Vivo* Imaging of Small Animals

Imaging was performed at 3, 24, and 72 h later of transplant with inhalational isoflurane anesthesia. We used the same incision in abdomen and inserted the probe into abdominal cavity (11). The imaging platform was a custom-built video-rate confocal fluorescence microscope. The probe was made of triplet graded index lenses with an OD of 1 mm and a stainless steel sleeve with an OD of 1.25 mm. After positioning our endoscope above the target tissue (i.e., cardiac graft, lymphoid tissues, and spleen), we moved the animal stage up while monitoring the real-time fluorescence images until the physical contact between the tissue and the lens probe was steady (7, 8). During the imaging session at cardiac graft, the surface of the graft normally moves up to 1–2 mm in both the vertical and lateral directions. This resulted in incomplete study with high resolution of imaging. However, the current system has an advantage compared with others in the use of beating-heart imaging because of suction attachment. A suction tube with a diameter of 2–3 mm stabilizes the local tissue motion safely and effectively at a suction pressure of 50 mm Hg, which resulted in favorable effects on local blood perfusion with cellular migration and exiting. We can control and change the dose of suction, and this suction surrounded the tip of camera so that cellular imaging of the beating heart *in vivo* is possible with minimal motion-induced artifacts. According to our imaging sessions, we indicated that local suction at 50–100 mm Hg causes minimal perturbations to the heart, both at the local tissue and at the organ levels (7, 8). To study the relative distribution or compartmentalization of dDC in the drainage lymph nodes (DLNs) of recipients *in vivo* in real time, we used our microscopy in different time points as described above. TAMRA dextran conjugate was intravenously injected as blood pool tracer.

## Results and Discussion

We previously showed that recipients of heart allografts from CX3CR1^−/−^ donors show longer survival (12). In addition, the same recipients with heart allografts from CX3CR1^−/−^ donors are resistant to the induction of tolerance by T cell costimulatory blockade, which indicated the importance of the CX3CR1 pathway in the generation of heart tissue DCs and the divergent role of tissue/DCs in rejection versus tolerance (12). CX3CR1^+^ cells were thought to be DC precursors that play an immunomodulatory role in the induction of tolerance. No data are yet available on the importance of chemokine pathways in regulating homeostasis of DC in heart transplants.

To better differentiate the chain of events underlying the body’s tolerance of transplanted tissue versus the events leading up to rejection, we employed the new powerful imaging tool to detect cell behaviors following transplant in the current study.

**Figure 2:**
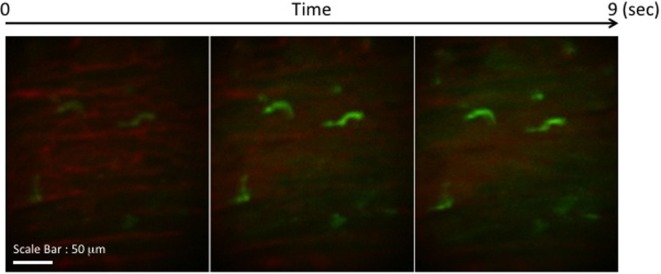
**Images of GFP^+^ cells in transplant model with using CX3CR1^GFP/+^ DC heart graft (24 h of posttransplant)**. CX3CR1^GFP/+^ DC heart graft into BALB/c recipient model, GFP^+^ cells were detected at cardiac graft. They stretched their shapes over time, but the shape was still roundish.

**Figure 3:**
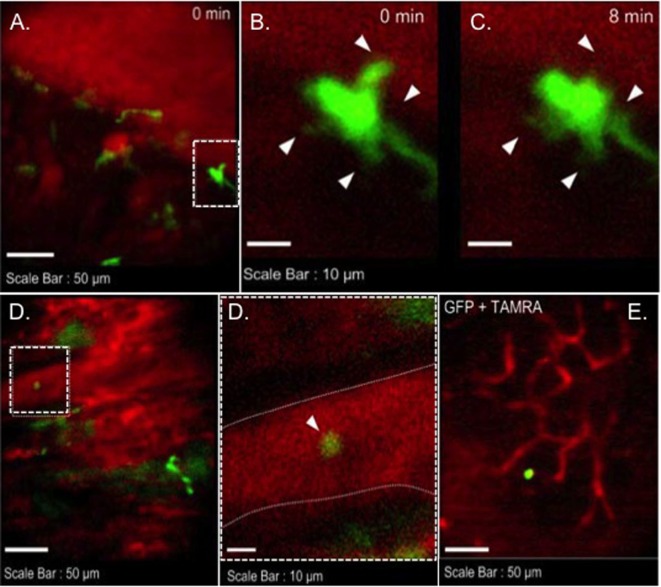
**Images of GFP^+^ cells in transplant model with using CX3CR1^GFP/+^ DC heart graft (72 h of posttransplant)**. CX3CR1^GFP/+^ DC heart graft into BALB/c recipient model, **(A–C)** shape change of GFP^+^ cell was observed in heart graft, **(D)** GFP^+^ cell in graft circulation (left panel) and vessel wall is marked by dotted line (right), **(E)** GFP^+^ cell image in mesenteric lymph node.

We transplanted CX3CR1^GFP/+^ DC hearts into complete MHC-mismatched BALB/c recipients. We performed several imaging sessions at 3, 24, and 72 h later of transplant under anesthesia. There were few significant changes in both 3 and 24 h imaging sessions (Figure 2; Video S1 in Supplementary Material); however, images from 72 h at cardiac graft showed like a “passengers at the airport.” GFP cells were trying to prepare for exiting from the graft (Figure 3; Videos S2–S5 in Supplementary Material). In addition, GFP^+^ cell motility and shape change was detected (triangle) in transplanted heart graft (Figures 3A–C). In contrast to earlier images of GFP^+^ cells (Figure 2), the morphology of GFP^+^ cell in the allograft was spiky and come to be a typical DC shape, which suggested that the first 3 days of posttransplant (24–72 h) may be a transition phase of DC maturation by allorecognition. In addition, we took images from the secondary lymphoid tissues to investigate further understanding of unsolved transplant immunological questions. Mice survive the procedure in our model, thereby permitting imaging of the same recipients between the beating cardiac allograft and secondary lymphoid tissues *via* the systemic circulation over time. Unfortunately, we almost failed to see donor DC at secondary lymphoid tissues by our IVM system. Only a few GFP^+^ cells were detected in the vasculature and lymph nodes (Figures 3D,E). From three lymph nodes imaged – two mesentric, one inguinal – only one GFP^+^ cell was observed (Figure 3E). Compared with same kind of experiment done by MHC class II GFP^+^ donor model, this is very small number (data not shown).

Since the maximum imaging depth of fluorescence microscopy is limited to 100 μm, the penetration depth is sufficient to access most of the major coronary vessels and capillaries and the myocardium in our experimental transplant model. However, there may be other immunological events in deeper tissues and areas where we cannot access to observe them with our IVM system. For the further investigation, we have already performed adoptive transfer models using colored recipient CD4 or CD8^+^ T cells to see cellular interactions between dDC and those T cells (Ueno et al., *American Transplant Congress* 2012, 2013, 2014, 2015). There is also a possibility that the CX3CR1 is not an optimal marker for the entire DC population in the heart. Furthermore, the lymph node investigated in this study may not represent the draining lymph nodes.

Based on advances in intravital imaging, it is now feasible to follow some of these key cellular and molecular events longitudinally in mice, which will provide us with an improved understanding of the highly dynamic temporospatial events from DC mobilization to activation, rather than a static snapshot provided by traditional experimental approaches.

## Author Contributions

TU and PK participated in the performance of the research, performed the data collection, performed the statistical analysis, and contributed to the writing of the manuscript; MM and MY participated in the writing of the manuscript and performed review; TS and KJ participated in the statistical analysis; MS and AC helped in the design of the study; and RA and SY participated in the review.

## Conflict of Interest Statement

The authors declare that the research was conducted in the absence of any commercial or financial relationships that could be construed as a potential conflict of interest.

## Supplementary Material

Video S1 **Live image of GFP^+^ cells at cardiac graft (24 h of posttransplant)**. GFP^+^ cells stretched their shapes over time, but the shape was still roundish (Lobby area).

Video S2 **Live image of GFP^+^ cells at cardiac graft (72 h of posttransplant)**. Roundish GFP^+^ cell showed up and started moving from right to left in myocyte (Ticketing).

Video S3 **Live image of GFP^+^ cells at cardiac graft (72 h of posttransplant)**. The cell was detected in a cardiac vessel (Security).

Video S4 **Live image of GFP^+^ cells at cardiac graft (72 h of posttransplant)**. The cell was still detected in a cardiac vessel (Duty Free).

Video S5 **Live image of GFP^+^ cells at cardiac graft (72 h of posttransplant)**. More GFP^+^ cells were detected at vessel areas (Boarding Gate) and some GFP^+^ cells already departed and were observed in a vessel.

## Abbreviations

dDC, donor dendritic cell; IVM, intravital optical microscopy.
